# Editorial: Towards an understanding of spinal and corticospinal pathways and mechanisms

**DOI:** 10.3389/fnhum.2023.1181647

**Published:** 2023-03-21

**Authors:** Leonardo Abdala Elias, Pilwon Hur, Gary C. Sieck

**Affiliations:** ^1^Department of Electronics and Biomedical Engineering, School of Electrical and Computer Engineering, University of Campinas, Campinas, SP, Brazil; ^2^Neural Engineering Research Laboratory, Center for Biomedical Engineering, University of Campinas, Campinas, SP, Brazil; ^3^School of Mechanical Engineering, Gwangju Institute of Science and Technology, Gwangju, Republic of Korea; ^4^Department of Physiology and Biomedical Engineering, Mayo Clinic, Rochester, MN, United States

**Keywords:** spinal cord, corticospinal pathways, neuromodulation, motor system, neurorehabilitation

Movement control involves a complex interplay of spinal and corticospinal mechanisms and pathways, providing flexibility for normal motor behaviors and plasticity after motor disorders. The parallel yet hierarchical organization of the motor system (Rothwell, 2012; Enoka, 2015) imposes challenges for designing methods and techniques to assess and modulate neuronal circuits since sensorimotor transformations are vastly distributed during reflexive, automatic, and voluntary actions. In this vein, the Research Topic aimed to bring about studies targeting the issue of effectively evaluating the function of spinal and corticospinal circuits involved in movement control under different physiological conditions, along with the alterations experienced by these neuronal circuits when neurorehabilitation/neuromodulation techniques are employed to recover/improve motor function. The eight studies presented in the Research Topic cover three main areas: neuromodulation of locomotor circuits, assessments of spinal cord excitability, and corticospinal activity in motor learning.

## 1. Neuromodulation of locomotor circuits

Locomotion is an automatic motor behavior whose intrinsic rhythmicity is provided by neuronal circuits within the brainstem and spinal cord (the so-called central pattern generators). These circuits, however, are highly adaptive, involving a large diversity of interneurons, and are influenced by sensory and cortical inputs (Kiehn, 2011; Enoka, 2015).

In Islam et al., stimulation of locomotor circuits by trans-spinal electrical stimulation decreased electromyographic activity of leg muscles of healthy and spinal cord injury (SCI) patients during stepping. However, disruption in motor coordination was observed only in the SCI group. Additionally, stimulation reduced spinal cord excitability in both healthy and SCI groups. Kawai et al., in turn, observed the emergence of distinct locomotion patterns (hopping- vs. walking-like) as the intensity of magnetic stimulation increased. Therefore, by adjusting the intensity of trans-vertebral magnetic stimulation, different subsets of interneuron circuits related to locomotor rhythms would be activated. Kaneko et al. combined functional electrical stimulation (FES) with active observation and motor imagery of walking movements to evaluate the influence of these techniques on corticospinal and spinal cord excitabilities of healthy participants. FES alone could not induce changes in either corticospinal or spinal cord excitabilities. The combined techniques enhanced corticospinal excitability in a dorsiflexor muscle but not in a plantar flexor. These studies show potential therapeutic approaches to induce locomotor-like movement and regulate the excitability of spinal and corticospinal pathways in neurological disorders affecting the locomotor circuits, such as stroke and SCI.

## 2. Changes in spinal cord excitability

The spinal cord is far from a simple relay of commands from the brain centers to the muscle. Instead, several intricate neuronal circuits and physiological mechanisms are embedded into the spinal cord and interact with descending commands providing a complex sensorimotor integration system that plays an essential role in movement control (Nielsen, 2016).

Batista-Ferreira et al. used the well-known H reflex to show increased spinal cord excitability during contraction in young and elderly participants, suggesting that the mechanisms behind the increase in reflex excitability would be independent of aging. A computational model of the neuromuscular system was employed to provide a putative explanation for the findings. The conclusion was that descending voluntary commands would decrease the threshold for reflex recruitment by depolarizing the membrane potentials of spinal motor neurons.

Barss et al. investigated the influence of upper limb vibration (a potential neurorehabilitation technique) on the activity of spinal and corticospinal pathways in healthy participants. The amplitude of motor-evoked potentials did not change with vibration, suggesting that corticospinal transmission is not altered compared to the no-vibration condition. Conversely, the authors reported that vibration inhibits the H reflex pathway and the medium latency response of the cutaneous reflex. The findings from conditioned (cutaneous stimulation) H reflexes support the hypothesis that presynaptic inhibition is enhanced by vibration, and this technique would help reduce spasticity in neurological patients.

Bunno and Suzuki explored how motor imagery of thenar muscles would influence the excitability of spinal motor neurons innervating the abductor digiti minimi (ADM) muscle of healthy participants. First, the authors showed that contraction of thenar muscles during pinching induced activation of ADM. Additionally, the persistence and relative amplitude of F waves from ADM were significantly increased during motor imagery of thenar muscles. Despite controversies on the validity of the F wave to assess motor neuron excitability (Espiritu et al., 2003; Pierrot-Deseilligny and Burke, 2012), the authors argued in favor of increased spinal excitability during motor imagery, reinforcing its potential as a neurorehabilitation approach.

## 3. Corticospinal excitability in motor learning

Motor learning represents a general term for diffuse adaptations along the motor system while acquiring and maintaining motor skills (Krakauer et al., 2019). It is frequently associated with a time-dependent improvement in some measures of motor performance (e.g., accuracy, variability, and promptness). Moreover, reducing motor variability is a hallmark of motor learning (Dhawale et al., 2017).

The studies by Macías et al. and Norup et al. explored how motor learning would affect the excitability of corticospinal pathways. In Macías et al., rodents engaged in a lever-pressing task while calcium fluorescence was synchronously measured in primary motor (M1) and somatosensory (S1) brain areas to infer the activity of the corticospinal pathway. After ~17 sessions, the animals learned to perform the task (reduced the reaction time and decreased trial-to-trial movement variability). Consequently, expert animals exhibited a significant increase in population calcium activity in both M1 and S1 in anticipation of movement, but M1 neurons sustained the increased activity during movement execution (lever pushing and release). In contrast, S1 neurons returned to basal activity just before starting the lever pressing. These results show the diversity in corticospinal tract function, with M1 and S1 exhibiting distinct temporal activities during motor learning. Norup et al. showed that the excitability of the corticospinal pathway in healthy humans significantly increased after practizing a position control (dynamic) task. Conversely, no change in corticospinal excitability was observed after the practice of a force control (static) task. The practice of either position or force control tasks improved accuracy. Learning a dynamic task resulted in accurate force control, while the reverse did not. In combination, these studies underline that sensorimotor integration in the motor cortex during motor learning is task-dependent and occurs in a non-homogeneous manner.

## Author contributions

LE wrote the first draft. LE, PH, and GS revised the text. All authors approved the final version of the editorial.
